# Exploring fibers with X-ray eyes

**DOI:** 10.1107/S2052252525011182

**Published:** 2026-01-01

**Authors:** Cinzia Giannini

**Affiliations:** a Istituto di Cristallografia Consiglio Nazionale delle Ricerche (CNR) Via Giovanni Amendola, 122/o 70126Bari Italy

**Keywords:** fibers, nanostructure, small-angle X-ray scattering, SAXS, wide-angle X-ray scattering, WAXS, materials science

## Abstract

The novel analytical approach for interpreting complex X-ray diffraction patterns from fibers described by Wang *et al.* [(2026), *IUCrJ*, **13**, 63–76] is discussed.

X-ray crystallography is a fundamental method, in materials science, for determining the atomic structures of crystalline materials (Giacovazzo, 2011 ▸). Among these materials, fibers hold a special place due to their inherent hierarchical order spanning sub-molecular and above-molecular scales, typically along two preferred orientations: the meridional direction (parallel to the fiber axis) and the equatorial direction (perpendicular to the axis) (Giannini, Olsson *et al.*, 2021 ▸). Natural fibers such as fibroin, elastin, DNA, type-1 collagen, silk and keratin are prominent examples from the biological world, possessing technologically relevant applications and significant impacts on the circular economy. Furthermore, fibers are clinically relevant in neurodegenerative diseases (*e.g.* Alzheimer’s or Parkinson’s disease), where fibrous proteins aberrantly assemble into larger, insoluble amyloid fibrils that accumulate in specific target organs (Ke *et al.*, 2020 ▸). In engineered contexts, synthetic materials – like self-assembled peptide bio-fibers – represent a new class of multifunctional materials fabricated through the precise assembly of amino-acid chains (Balasco *et al.*, 2021 ▸).

In all these instances, bio-fibers – whether natural or man-made – are semi-crystalline materials featuring crystalline nanodomains embedded within an amorphous (or highly disordered) matrix and organized hierarchically. This intrinsic organization frequently renders them unsuitable for traditional crystallographic methods. Instead, their analysis necessitates more tailored solutions that rely on X-ray scattering techniques, such as small- and wide-angle X-ray scattering (SAXS, WAXS) to explore above- and sub-molecular order, respectively. In Fig. 1 ▸, WAXS and SAXS data collected on type-1 collagen fibers of an equine tendon tissue are shown (Giannini, De Caro *et al.*, 2021 ▸).

In the current issue of *IUCrJ*, Wang *et al.* report the development of a novel *analytical* approach for interpreting complex X-ray diffraction patterns from fibers (Wang *et al.*, 2026 ▸). For model validation, the authors investigated the ultrastructure of the stomatopod tergite cuticle. This sample was chosen because its known Bouligand plywood architecture of chitin fibrils provides a well characterized benchmark in existing literature (Zimmermann *et al.*, 2013 ▸).

Traditionally, interpreting X-ray patterns from fiber-like structures requires assuming that samples are well aligned. Real biological tissues, however, are rarely so ordered. They are hierarchical, textured and often incorporate controlled imperfections that enhance mechanical performance (Meyers *et al.*, 2008 ▸; Wegst *et al.*, 2015 ▸; Fratzl & Weinkamer, 2007 ▸). What Wang and colleagues have done is develop a theoretical method that explicitly incorporates this natural imperfection and turns it into meaningful structural information.

Their analytical framework provides a generalized description of how X-rays scatter from partially oriented crystallites, capturing the full angular dependence of diffraction arcs. In simpler terms: the model accounts for how fibrils tilt, rotate and vary in orientation around a central axis. This is particularly valuable when analyzing helicoidal or ‘twisted plywood’ structures, characteristic of many fibers (Bouligand, 1972 ▸).

This innovation is particularly valuable for understanding the mantis shrimp’s dactyl club. The club’s helicoidal arrangement of chitin fibrils – present in specific subsurface regions of the impact surface – grants the animal its extraordinary resistance to repeated high-energy strikes. Yet when X-rays interact with such a twisted architecture, the resulting diffraction pattern becomes extremely complex: individual reflections smear, rotate and warp depending on fibril orientation and the local twist angle. Prior to this work, reconstructing these features typically required computationally intensive numerical simulations or oversimplified models that were unable to capture the full geometric complexity.

The key achievement of Wang *et al.* is to derive closed-form analytical expressions describing how diffraction rings appear when the sample is only partly aligned. These expressions establish a quantitative relationship between fibril orientation distributions and the intensity pattern on the detector. Importantly, the method allows the angular distribution function of the fibrils to be determined, enabling researchers to quantify both the average structure and the degree of orientational disorder.

To demonstrate the power of the approach, the authors apply it directly to wide-angle X-ray diffraction data from mantis shrimp cuticle. The model captures subtle features – such as asymmetric arc intensity, variations in ring thickness and non-uniform angular broadening – that reflect local helicoidal twists and fibril misalignments. These aspects were notoriously difficult to interpret using traditional diffraction theory. The fact that the authors achieve this through an analytical (rather than numerical) model is particularly noteworthy: analytical expressions allow faster fitting, more intuitive parameter interpretation and easier generalization to other systems. For experimentalists working with synchrotron or laboratory X-ray sources, this can dramatically speed up data analysis and make previously inaccessible information available.

The impact of this work goes far beyond a single biological example. Many biological and synthetic materials – collagen, cellulose, keratin, silk, muscle fibers, nanotube bundles, man-made polymeric fibers – share this type of partial alignment. Until now, researchers had to rely on simplistic assumptions that limited the precision of their structural interpretations. Having a mathematically rigorous description of diffraction from such systems opens the door to a new generation of quantitative analyses.

From a broader perspective, this work is a reminder of how much information is hidden in the way X-rays scatter off matter. The patterns recorded on a detector, decoded with the right mathematical tools, become a map of the internal world of materials. When those tools are refined, as in this study, our ability to read that map deepens.

The study also aligns with a growing movement in materials science: the desire to understand and harness complex hierarchical structures. Nature rarely builds with perfect crystals; instead, it uses gradients, twists and controlled disorder to achieve exceptional properties. The new analytical framework allows scientists to quantify these features with unprecedented clarity.

Looking forward, one can imagine multiple applications. Biomimetic materials designed to replicate the toughness of crustacean shells. Advanced composites where fibers are deliberately misaligned to improve impact resistance. Real-time studies of how fibrils orient during 3D printing or processing of polymer solutions. Even the structural characterization of viral capsids or soft biological tissues could benefit, just to cite few examples.

In essence, this work helps to bridge the gap between idealized models and the rich complexity of real materials. It provides a language – mathematical, but accessible to practitioners – to describe the subtle interplay between order and disorder that makes biological fiber systems so effective. As interest grows in designing engineered fibrous materials inspired by nature, such analytical tools will be indispensable.

## Figures and Tables

**Figure 1:**
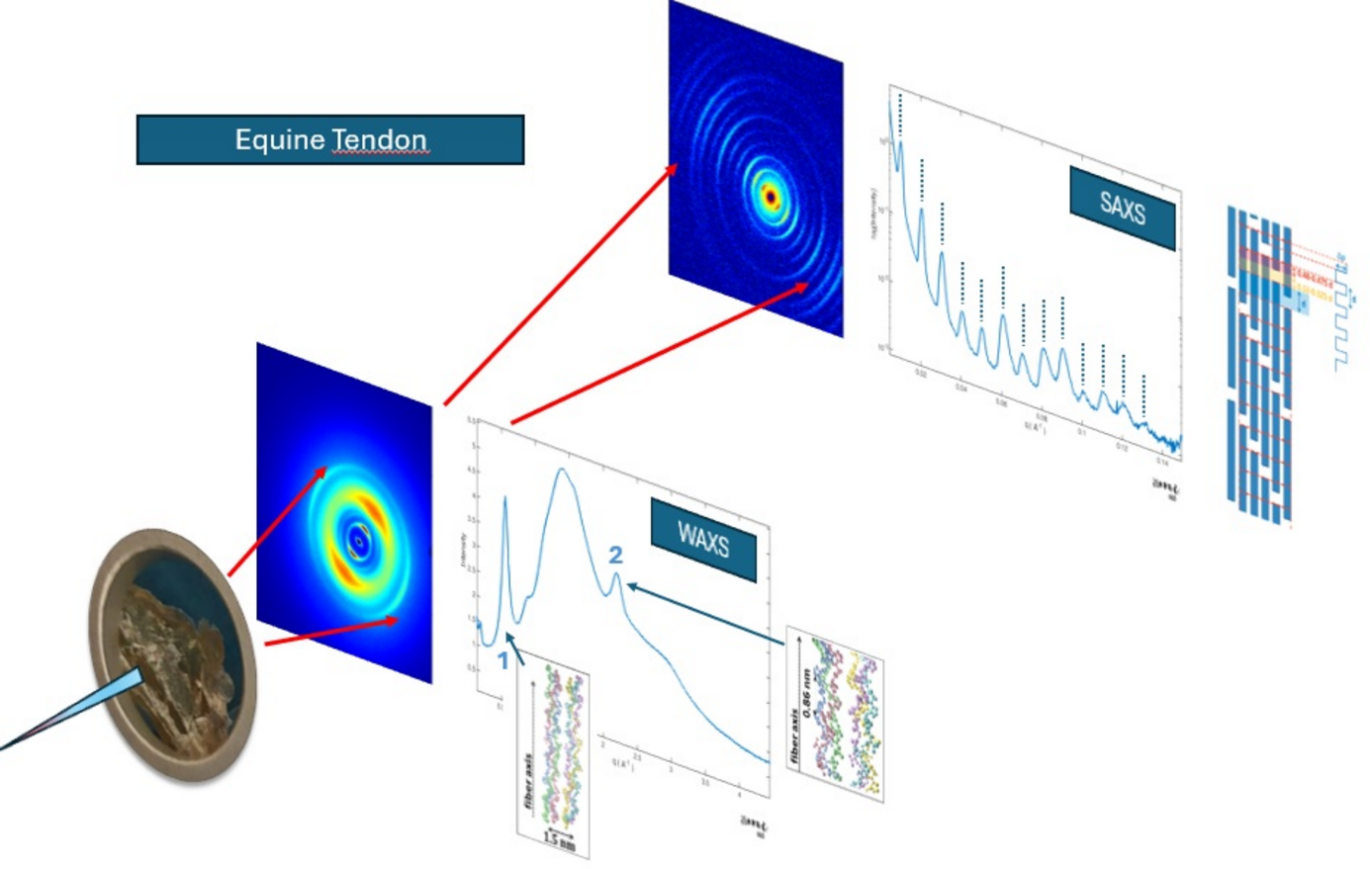
WAXS and SAXS 2D and 1D data collected on type-1 collagen fibers of equine tendon tissues. The equatorial (1) and meridional (2) WAXS peaks as well as the entire series of SAXS diffraction orders (marked with dotted vertical bars) correspond to the sub-molecular and above-molecular hierarchical structure, as detailed in Giannini, De Caro *et al.* (2021 ▸).
